# Environmental Pollutants and Metabolic Disorders: The Multi-Exposure Scenario of Life

**DOI:** 10.3389/fendo.2018.00582

**Published:** 2018-10-02

**Authors:** Brigitte Le Magueresse-Battistoni, Hubert Vidal, Danielle Naville

**Affiliations:** Univ Lyon, CarMeN Laboratory, INSERM U1060, INRA U1397, INSA Lyon, Université Claude Bernard Lyon1, Oullins, France

**Keywords:** cocktail effect, mixture, endocrine disrupting compounds (EDCs), pollutants, sex-dimorphism

## Abstract

Obesity and diabetes have reached epidemic proportions the past few decades and continue to progress worldwide with no clear sign of decline of the epidemic. Obesity is of high concern because it is the main risk factor for a number of non-communicable diseases such as cardiovascular diseases and type 2 diabetes. Metabolic diseases constitute a major challenge as they are associated with an overall reduced quality of life and impose a heavy economic burden on countries. These are multifactorial diseases and it is now recognized that environmental exposure to man-made chemical pollutants is part of the equation. Yet, risk assessment procedures are based on a one-by-one chemical evaluation which does not meet the specificities of the multi-exposure scenario of life, e.g., a combined and long-term exposure to even the smallest amounts of chemicals. Indeed, it is assumed that environmental exposure to chemicals will be negligible based on the low potency of each chemical and that they do not interact. Within this mini-review, strong evidences are brought that exposure to low levels of multiple chemicals especially those shown to interfere with hormonal action, the so-called endocrine disrupting compounds do trigger metabolic disturbances in conditions in which no effect was expected if considering the concentration of each individual chemical in the mixture. This is known as the cocktail effect. It means that risk assessment procedures are not protective enough and thus that it should be revisited for the sake of Public Health.

## Introduction

Obesity and diabetes have reached epidemic proportions the past few decades and continue to progress worldwide with no clear sign of decline of the epidemic in any country. An estimate of 1.9 billion adults were overweight in 2016 of these over 650 million were obese. Children and teenagers are also of high concern with 380 million overweight or obese in 2016. Latest projections by WHO (World Health Organization) indicate that proportion of overweight and obese males and females will continue to increase and reach 3.3 billion by 2030 (1). Obesity is of high concern because it is the main risk factor for a number of non-communicable diseases such as cardiovascular diseases, certain cancers and type 2 diabetes. Indeed, almost 90% of persons suffering from type 2 diabetes are obese and more than 400 million persons will suffer from diabetes by 2030. Metabolic diseases constitute a major challenge as they are associated with an overall reduced quality of life, psychological problems and several physical disabilities (1, 2). Metabolic diseases also impose a heavy economic burden on countries. It is a major cause of death and it costs an average of $ 2000 billion to the global economy, almost 3 points of GDP (gross domestic product) (3, 4).

Metabolic diseases are multifactorial diseases. Apart from genetic susceptibility, life-style risk factors associating over-nutrition and sedentary behavior are major contributors. Yet these causative factors do not explain the magnitude of metabolic diseases or the kinetics of the epidemic. Among other etiologic factors, it is acknowledged that environmental exposure to man-made chemical pollutants is part of the equation, especially those shown to interfere with hormonal action, the so-called endocrine disrupting compounds (EDCs) (5–12).

Today's non-occupational exposure to chemicals is characterized by exposure to tens of thousands of man-made chemicals at low levels. Occupational exposure will not be considered in this mini-review. Another characteristic of non-occupational exposure is its chronicity, from conception onwards thus encompassing gestation and lactation which are periods highly vulnerable to chemicals. It is indeed recognized that threat during the maternal period (e.g., food restriction, chemical stressors) could trigger diseases later in life including metabolic diseases and some cancers, known as the Developmental Origin of Human adult Diseases (DOHaD) concept (13, 14). In addition, the nature of chemicals and doses to which individuals are exposed may vary across their lifespan. It is also of concern that the number of chemicals to which humans are exposed has continued to grow for more than 100 years as is their spatial distribution while the identification of their possible hazardous effects lags behind. Furthermore, exposure is non-deliberate and the exposed population is seldom aware of the nature of the chemicals, the levels to which it is exposed as well as the health consequences. Hence, it appears that risk assessment procedures need to be revisited because the one-by-one evaluation of chemicals does not meet the specificities of the multi-exposure scenario of life.

Within this mini-review we aim in presenting basic characteristics on environmental pollutants including EDCs and risk assessment principles operating nowadays. We will next summarize evidences gathered using either natural or artificial mixtures in experimental models, that exposures to low levels of multiple chemicals trigger metabolic disorders in conditions in which no effect was expected if considering the concentration of each individual chemical in the mixture. This is defined as the cocktail effect of pollutants which certainly constitutes one of the biggest health challenges in our modern societies.

## Risk assessment evaluation principles and limits

If industrialization has promoted societal progress improving life expectancy, it also led to the presence of tens of thousands of anthropogenic chemicals transported in the atmosphere and globalizing pollution. Man-made sources of pollution are related to industrial and agricultural activities but also to domestic activities. Pollutants are found in foods, beverages and packaging, clothes, cosmetics and cleaning products, furniture, paints, electronic equipment, plastics, and the list is long. Routes of exposure are mostly through diet but also dermal and by inhalation not to mention transfer via placenta, suckling and mouthing behavior placing babies and small infants at particularly high risk for they lack a mature defensive xenobiotic detoxification system. Pollutants differ according to their natural disposal in degradable and low-degradable or persistent pollutants including the lipophilic persistent organic pollutants (POPs) which bio-accumulate through the food chain in fatty tissues with half-lives of several years in humans (15). Although severely regulated because of environmental toxicity (e.g., the ban of polychlorobiphenyls, PCBs, in the 1970's), POPs still contaminate air, soil and water compartments. Other pollutants may be more quickly degradable, particularly those produced by the plastic industry (phthalates and bisphenols) but because they are produced at very high volumes and massively found in daily consumables they are consistently detected in human body fluids such as plasma or urine, thus reflecting substantial and continuous exposure (16, 17). However regulatory bodies, e.g., the U.S. Environmental Protection agency (EPA) or the European Food Safety Agency (EFSA), do not consider combined exposures in risk assessment procedures or long-term exposure to even the smallest amount of chemicals. Exposure limits are setup for each individual chemical with the definition of reference doses such as the Tolerable Daily Intake (TDI). TDIs are extrapolated from the no or low observed adverse effect level (NoAEL/LoAEL) in experimental studies assuming linearity of the effects and therefore a threshold under which effects will be negligible. With this assumption (which does not apply to genotoxicant carcinogens for which there is no threshold), it can be deduced that risks arising from environmental exposure to chemicals in a real life exposure scenario will be negligible based on the low potency of each chemical of the mixture. But this assumption excludes several aspects linked to environmental exposure and related to the concept of threshold and the supposed linearity of the adverse effects not to mention the effects at low doses. By low doses it is meant environmental doses or concentrations found in biological fluids or doses approaching toxicological reference values (18). This indicates that risk assessment procedures may not be protective enough when it comes to environmental exposure to chemicals and more specifically to EDCs.

## EDCs and some of their characteristics

EDCs are exogenous substances or mixture of chemicals that interfere with any aspect of hormone action, i.e., EDCs can mimic or antagonize hormonal action and interfere with the mechanism of hormonal production, transport or metabolism (12). Energy homeostasis is one of the multiple physiological functions controlled by the endocrine system and as such a target of EDCs. It depends on the integrated action of various hormones including insulin, leptin, growth hormone, thyroid hormones, glucocorticoids but also sex hormones. Importantly, the hormonal interplay intricacies and the nature of the hormones at stake vary with age and sex and the state of maturity of the endocrine function of concern. For example, regulation of metabolism is highly age- and sex- dependent e.g., feeding behavior, distribution of fat masses and insulin sensitivity (19). Thus, considering the intrinsic properties of the endocrine system and the definition of the EDCs based on their mode of action and not their chemical structure, the important parameters that should be considered in risk assessment evaluation are: (1) the dose and the non-linearity of the induced effects, (2) the timing and the length of exposure and (3) the simultaneous presence of several chemicals in mixture (18, 20, 21). Additionally, it is emphasized that the less an endocrine function is mature at the time of chemical exposure, the more dramatic health adverse effects will happen defining highly vulnerable periods such as gestation and lactation. For example, ancestral exposure of mice to obesogen chemicals such as organotin tributyltin (TBT) was found to predispose unexposed descendants to obesity (22).

About a thousand of chemicals could display EDC activities (23) and a subset was identified as metabolic disruptors because they favor/aggravate obesity and/or insulin resistance leading to diabetes. Metabolic disruptors may include Bisphenol A (BPA)and dichlorodiphenyltrichloroethane (DDT) but also certain pesticides, perfluorinated compounds and phthalates in addition to TBT. They can target the signaling pathways of different nuclear receptors including the steroid receptors, but also xenobiotic receptors or receptors activated by peroxisome proliferators, PPAR (peroxisome proliferator-activated receptor) and its heterodimeric partner retinoid X receptor (RXR), not to mention non-genomic signaling pathways and cross-talk with the various nuclear receptors (24–27).

## Evidences for a cocktail effect from natural or artificial mixtures on metabolic disruption and its sex-dimorphism

First evidences of a cocktail effect resulting from exposure to environmental mixtures were brought by scientists invested in Biology of Reproduction with the demonstration of additive effects in mixtures containing chemicals sharing a similar mechanism of action and combined in mixtures at concentrations that individually do not result in observable adverse effects. This was called the something from “nothing” phenomenon demonstrated originally with a mixture of 8 weak estrogenic chemicals (28) but also using combinations of anti-androgens (29) or more complex mixtures of anti-androgenic pesticides, antioxidants, industrial pollutants and chemicals present in personal care products (30). The concept of additivity was also the basis of the Toxic Equivalent Factor (TEF) set up by the WHO (31) to facilitate risk assessment of “natural” mixtures of dioxins, furans and dioxin-like PCBs, using the dioxin of Seveso (named after the industrial explosion in the village of Seveso, Italy), the most potent in activating the aryl hydrocarbon receptor (AhR), as reference.

First demonstrations of mixture effects in the metabolic disruption field area emerged less than 10 years ago (Table 1) when it was observed that rats fed refined salmon oil for 28 days exhibited better metabolic outcomes than rats fed crude salmon oil (32). These experiments suggested that exposure to POPs commonly present in food chains could trigger enhanced body weight and visceral fat, liver steatosis, glucose intolerance and insulin resistance (32) as well as chronic low-grade inflammation in adipose tissue (33). A few years later, we brought (34) the proof-of-concept study that a mixture of low-dosed pollutants not sharing similar mechanisms of action, could trigger metabolic disturbances in mice. Our original model was designed to take into consideration several parameters of the real life including chronic exposure at low doses covering all developmental stages from the fetal period to adult onwards. It even started when dams were immature females of 5 weeks of age. Both male and female mice (and not only adult males as classically studied) were evaluated to explore the sex-biased mechanisms linked to endocrine disruption. The pollutant cocktail was incorporated in either a high-fat high-sucrose (HFHS) diet (34) or a standard diet (37) to reflect different nutritional situations also considering that food is a primary route of exposure. Thus, we selected pollutants known to contaminate food and not sharing similar mechanisms of action to better reflect a “real” scenario of exposure. The mixture was made of two persistent pollutants including the most powerful dioxin (2,3,7,8-Tetrachlorodibenzo-*p-*dioxin, TCDD) and the most abundant of the non-dioxin like PCBs (PCB153). The other two were short-lived pollutants including BPA, one of the substances most investigated for its endocrine disrupting activities (18, 42, 43) and the di-[2-ethylhexyl] phthalate (DEHP) largely used to soften plastics. Each chemical in the mixture was used at a dose in the range of its tolerable daily intake (TDI). With regards to their modes of action, these well-recognized EDCs display mostly estrogeno-mimetic and anti-androgenic activities for BPA and DEHP, respectively, while dioxins interact with AhR and non-dioxin like PCBs may interact with other xenobiotic receptors such as constitutive androstane receptor (CAR) and pregnane X receptor (PXR) (18, 24, 44, 45). Moreover, interaction of pollutants of the mixture may also occur with other receptors like thyroid receptors or glucocorticoid receptors not to mention extensive cross-talks which may occur via direct or indirect interaction with metabolic pathways regulating energy homeostasis as reviewed elsewhere (8, 24). With such a model, we originally demonstrated that the metabolic adverse impact in mice exposed to the mixture lifelong occurs in the absence of any weight gain but was strongly sex-dependent (34, 46), and also related to the age of the animals (47) and their nutritional environment (37). The female offspring exposed to the mixture incorporated in a HFHS diet exhibited aggravated glucose intolerance, impaired estrogen signaling in liver and enhanced expression of inflammatory markers in the adipose tissue at adulthood as compared to non-exposed females. This suggested that pollutants could lessen the protection of estrogens against the development of metabolic diseases. Importantly, these effects were not observed in younger females or in males which are characterized by different hormonal milieu in line with endocrine disrupting effects. Males had impaired cholesterol metabolism resulting from enhancement of genes encoding proteins related to cholesterol synthesis and degradation in bile salts when fed a HFHS diet containing the mixture of pollutants (34, 47). The observed metabolic impact in females was dependent on the nutritional context as female mice fed a standard diet and exposed similarly to the mixture showed alteration of lipid homeostasis with no difference in body weight or glucose tolerance (37). Importantly, pollutants elicited distinct and common features as compared to a HFHS diet as revealed by a comparative hepatic transcriptomic study. Among features resulting from pollutant exposure in the liver was the finding of several dysregulated genes belonging to the circadian clock metabolic pathway including major canonical genes of the core clock (*Period circadian regulators 1-3, Arntl1* encoding BMAL1 and *Clock*) and clock regulators thus highlighting circadian disruption (37). None of the described effects observed in females were described in males pointing to the sex-dimorphic impact of pollutants consistent with the sex-dimorphic regulation of energy homeostasis (48) (Table 1).

**Table 1:** Metabolic effects of pollutants in mixture and sex-dimorphism.

**Mixture of pollutants**	**Composition**	**Animal model**	**Metabolic effects of the mixture**	**References**
Natural mixture: crude or refined salmon oil in high-fat (HF) diet	mixture of Persistent Organic Pollutants (POPs): organochloride pesticide, Dichlorodiphenyltrichloroéthane (DDTs), dioxins, Polychlorobiphenyls (PCBs)	Adult male Sprague-Dawley rat (28 days of exposure)	HF+ crude oil vs HF: insulin resistance, abdominal obesity, liver steatosis, down-regulation of genes involved in lipid homeostasis (Insig-1 and Lipin 1) in liver	Ruzzin et al. (32)
Very high fat diet (VHF) or Western diet (WD) containing farmed salmon filet (VHF/S and WD/S)	mixture of Persistent Organic Pollutants (POPs): organochloride pesticides, dioxins, furans and Polychlorobiphenyls (PCBs)	8-weeks old male C57BL/6J mice (8 weeks exposure for VHF; 6 weeks for WD)	VHF/S *vs* VHF: aggravation of insulin resistance, visceral obesity and glucose intolerance, adipose tissue inflammation. Increased blood glucose and plasma insulin. WD/S *vs* WD: enhanced body weight, overgrowth of adipose tissue, increased glucose intolerance and insulin resistance with increased plasma insulin	Ibrahim et al. (33)
Combination of four Endocrine Disrupting Chemicals (EDCs) in high fat-high sucrose (HFHS) diet	2,3,7,8-Tetrachlorodibenzo-p-dioxin (TCDD), Polychlorobiphenyls (PCB) 153, Bisphenol A (BPA), di (2-ethylhexyl) phthalate (DEHP) each at reference doses (Tolerable Daily Intake, TDI for Human)	Male and female C57Bl/6J mouse (exposure from pre-conception, gestation to 12 weeks of life)	Sex-dependent metabolic disorders in the absence of weight gain. In males, increased hepatic expression of genes encoding proteins related to cholesterol biosynthesis associated with a decrease in hepatic total cholesterol levels. In females, marked deterioration of glucose tolerance associated with decreased expression of gene encoding ERα as well as estrogen target genes and increased expression of gene encoding the estrogen sulfotransferase SULT1E1.	Naville et al. (34)
Combination of Endocrine Disrupting Chemicals (EDCs) administered intragastrically to the exposed group.	Combination of di (2-ethylhexyl) phthalate, DEHP (15 mg/kg bw) with a mixture of Polychlorobiphenyls, PCBs (Aroclor 1254 at 7.5 mg/Kg bw/day)	female and male mice (12 days of exposure)	Increased liver weight but no difference in body weight; in liver, increased expression of gene encoding Peroxisome Proliferator-Activated Receptor (PPAR)^γ^ (males and females), decreased expression of genes encoding Estrogen Receptor (ER)α and phospholipase A (PLA) only in males	Lin et al. (35)
Different combinations of 3 pollutants (2 by 2 or all 3)	Nonylphenol (NP), tert-octylphenol (t-OP), Bisphenol A (BPA) (5 mg/kg bw/day of each)	Juvenile seabream (21 days)	Hepatic steatosis, modulation of the expression of genes involved in lipid metabolism (ppars, lpl, fasn, hsl) mostly using NP+t-OP or BPA+NP. Effects milder than those obtained with one chemical (hypothesis of possible interactions among compounds).	Carnevali et al. (36)
Combination of four Endocrine Disrupting Chemicals (EDCs) in low fat diet	2,3,7,8-Tetrachlorodibenzo-p-dioxin (TCDD), Polychlorobiphenyls (PCB) 153, Bisphenol A (BPA), di (2-ethylhexyl) phthalate (DEHP) each at reference doses (Tolerable Daily Intake, TDI for Human)	female C57Bl6/J mouse (exposure from pre-conception, gestation to 12 weeks of life)	Alteration of lipid homeostasis (increase of hepatic triglycerides) with no difference in body weight or glucose tolerance. Transcriptome analysis in liver highlights dysregulation of genes involved in fatty acid/lipid and circadian clock metabolic pathway. Most of these effects were observed in females and not in males.	Labaronne et al. (37)
Mixture of five prevalent organochlorine pesticides or their metabolites and five Polychlorobiphenyls (PCBs) present in contaminated salmon (32)	Dichlorodiphenyldichloroethylene (p,p'DDE); Dichlorodiphényldichloroéthane (p,p'DDD); hexachlorobenzene, dieldrin, trans-nonachlor, PCB-153, PCB-138, PCB-118, PCB-77, PCB-126 (oral gavage twice weekly during 7 weeks)	5 week-old male wild type C57Bl/6J and male ob/ob mice	Alteration of systemic lipid metabolism in ob/ob mice: increased hepatic triglycerides (TG) with decrease of serum TG levels (no difference either in plasma glucose or insulin levels or in inflammation in liver or adipose tissue). Induction of the expression of *Cyp3a11* in WT mice not in ob/ob mice	Mulligan et al. (38)
Mixture of 13 chemicals	Carbaryl, dimethoate, glyphosate, methomyl, methyl parathion, triadimefon, aspartame, sodium benzoate, Ethylenediaminetetraacetic acid (EDTA), ethylparaben, butylparaben, BPA, acacia gum (6-month exposure in drinking water at three different doses: low, medium and high).	8-week old Female and Male Sprague-Dawley rats	Increased body weight and alteration of hepatotoxic parameters (increased level of total bilirubin, alanine aminotransferase and alkaline phosphatase) even at low dose and only in males. Increased catalase activity with the low doses both in males and females. Also evidence for sex differences in some markers of the redox status (catalase levels, protein carbonyls).	Docea et al. (39)
Mixture of 4 fungicides and 2 insecticides in standard diet	Ziram; Chlorpyrifos; Thiacloprid; Boscalid; Thiofanate; Captan (52 weeks of exposure; at Tolerable Daily Intake, TDI for Humans)	16-week-old female and male C57BL/6J (WT) and Constitutive Androstane Receptor (CAR)-invalidated mice	In Wild Type (WT) males: increased body weight (not seen in male CAR-/-) and adiposity, hepatic steatosis, fasting hyperglycemia and strong glucose intolerance. In WT females: fasting hyperglycemia and slight glucose intolerance. Pesticide-exposed CAR-/- females exhibited pesticide toxicity with increased body weight and mortality rate. Sexually dimorphic alterations of various metabolic pathways	Lukowicz et al. (40)
Pesticide mixture containing six chemicals at 3 different doses (noted 5–16–37.5%)	Cyromazine, MCPB (4-(4-chloro-2-methylphenoxy) butanoic acid), Pirimicarb, Quinoclamine, Thiram, Ziram (daily oral gavage of pregnant rats from gestation day 7 to pup day 16)	Wistar rats - Male and Female offspring studied until 15 weeks of life	Decreased body weight at birth for both sexes with the highest dose. No difference in body weight after 15 weeks. Differences observed between males and females for several regulatory factors (such as leptin).	Svingen et al. (41)

Other combinations of low-dosed pollutants in mixture have later been tested in mouse (35, 38, 40) or rat (41) models as well as in juvenile seabream (36). These studies also reported significant alterations of the hepatic gene signature consistent with the liver being the primary site for detoxification (Table 1). Interestingly, whenever males and females have been studied, sex-differences have also been observed (35, 40, 41) as compiled (Table 1) which highlights the necessity, as mandated by the National Institutes of Health (NIH), to consider sex as a biological variable (49). Also consistent with our data (34, 37), it was shown that the nutritional component was as well a variable to consider. Indeed, exposure to a mixture of POPs (38), to dioxins (50) or to a pharmaceutical drug cocktail (51) resulted in differential metabolic responses between lean and obese mice. Outcomes surveyed included hepatic steatosis and systemic lipid metabolism (38), hyperglycaemia and hepatic mitochondrial function (51) and hepatic fibrosis (50). Whether this is linked to substantial alterations of the expression of hepatic xenobiotic processed genes (37, 52), resulting in differential ability of the liver to detoxify chemicals, warrants further studies.

## Conclusions

The current legislation on chemical risk assessment is definitely obsolete and needs to be updated to take into account the cocktail effect of mixtures. This is certainly a major challenge for the near future as environmental pollutants are risk factors for numerous pathologies also including cancers and hormono-dependent cancers among them, for which obesity is a risk factor (7). Importantly, it was assumed that the carcinogenic potential of chemical mixtures may be more important than that of individual carcinogens in the real world (53). It will also be critical to enhance our knowledge on chemical toxicity as according to the US EPA, it is available for less than 20% of substances produced at significant amounts, worldwide (54). While additivity was demonstrated for mixture of low-dosed chemicals affecting similar outcomes (55), when it comes to mixtures with molecules acting differently, which better fits the real life scenario, the problem becomes more complex because chemical actions may (41, 56) or may not be independent. For example, in an *in vitro* model of mesenchymal cells, BPA, DEHP and TBT could not be deduced from single compound experiments (57). As well synergistic activation of human PXR was observed in an *in vitro* model of hepatocytes (HepG2) by binary cocktails of pharmaceutical and environmental compounds (58). Another limitation to the additivity concept of cocktail effect lies in the fact that a pollutant can alter the metabolism of another pollutant present in the mixture and potentially its bioavailability (59). Moreover, some products may, by modifying the epigenome, leave an imprint on unexposed generations as recently demonstrated with TBT on RXR activation (60).

The problem posed by the cocktail effect is a virtually insurmountable challenge but it will have to be overcome for the sake of public health. Political authorities should work to reduce the exponential production of industrial chemicals. In addition, integrative approaches combining knowledge gathered in epidemiologic and biomonitoring studies, but also experimental, *in vitro* and *in silico* studies, together with computational approaches to construct predictive models, will certainly help at moving a path forward.

## Author contributions

The manuscript was written by BLM. DN created the Table and participated in the editing of the manuscript. HV participated in the editing of the manuscript. All authors approved the final version of the manuscript.

### Conflict of interest statement

The authors declare that the research was conducted in the absence of any commercial or financial relationships that could be construed as a potential conflict of interest.
